# The Critical Role of Empathy Over Dominance/Nurturance in Predicting Sexual Aggression in the Confluence Model

**DOI:** 10.1007/s10508-026-03510-5

**Published:** 2026-09-04

**Authors:** Philipp Yorck Herzberg, Hendrik Brunner, Swetlana Wildfang, Stefan J. Troche

**Affiliations:** 1 Department of Personality Psychology and Psychological Assessment, Helmut-Schmidt-University/University of the German Federal Armed Forces Hamburg, Holstenhofweg 85, 22043 Hamburg, Germany; 2 Europäische Fernhochschule Hamburg, Hamburg, Germany; 3 Institut für Psychologie, Universität Bern, Bern, Switzerland

**Keywords:** Confluence model of sexual aggression, Hostile masculinity, Impersonal sexuality, Empathy, Response surface analysis

## Abstract

The confluence model of sexual aggression identifies hostile masculinity and impersonal sexuality as core risk factors, with their interaction amplified in men high in dominance and low in nurturance; empathy has been proposed as the mechanism underlying this moderation. The present study tested whether empathy accounts for the effects of dominance/nurturance or whether dominance/nurturance contributes unique variance beyond empathy. In an online sample of 300 heterosexual men (age 18–65 years), sexual aggression was assessed with the Sexual Experience Survey, hostile masculinity with the Hostile Sexism Scale of the Ambivalent Sexism Inventory, impersonal sexuality with the Sociosexual Orientation Inventory Revised, dominance/nurturance via the difference score of the Bem Sex-Role Inventory Revised, and empathy with the Interpersonal Reactivity Index. Three latent negative binomial regression models were compared using structural equation modeling. Hostile masculinity and impersonal sexuality consistently predicted higher sexual aggression counts; empathy was a significant negative predictor, whereas the dominance/nurturance difference score was not. No latent interactions reached significance, but complementary response surface analysis confirmed a synergistic hostile masculinity × impersonal sexuality effect. Results indicate that empathy, not dominance/nurturance, attenuates sexual aggression risk within the confluence model. Findings recommend replacing dominance/nurturance with empathy in future confluence model research and prioritizing empathy-enhancing components in prevention programs targeting high-risk men.

**Supplementary Information:**

The online version contains supplementary material available at https://doi.org/10.1007/s10508-026-03510-5.

## Introduction

Sexual aggression of men toward women is a deeply rooted societal problem (World Health Organization, 2021). It manifests in subtle forms as well as extreme forms. The heterogeneity of sexually aggressive behaviors complicates a uniform definition (Bouffard & Goodson, 2017). However, a common denominator might be seen in Krahé et al., (2014) proposal that sexual aggression refers to behavior that intends or results in making another person engage in sexual activity, although the person is not willing to do so.

As an explanatory framework of sexual aggression, Malamuth developed the confluence model of sexual aggression across the last 40 years (Malamuth & Hald, 2017; for a review, see Bruera et al., 2023). This model postulates that two characteristics particularly contribute to increasing the risk of committing sexually aggressive acts. These two key characteristics are hostile masculinity and impersonal sexuality, which have been extensively investigated in prior research (Malamuth, 1998, 2003; Malamuth & Hald, 2017; Malamuth et al., 1991, 2021). According to Malamuth and Hald (2017), the broad construct of hostile masculinity combines two components: “(a) a narcissistic, insecure, defensive, hypersensitive, and hostile-distrustful orientation, particularly toward women, and (b) sexual gratification from controlling or dominating women” (p. 54). In line with this definition, Ray and Parkhill (2021) review corroborates that hostility toward women and sexual dominance are two major operationalizations of hostile masculinity in empirical studies. However, within Malamuth and Hald’s first component, Ray and Parkhill further differentiate between adversarial sexual beliefs, rape myth acceptance, and acceptance of interpersonal violence. From a development perspective, it has been assumed that environments characterized by adherence to traditional masculinity ideologies might cultivate and perpetuate such negative attitudes toward women (Murnen, 2015). Men growing up in such environments might be susceptible to experiencing gender-role stress, prompting compensatory behaviors aimed at reasserting their perceived masculinity. One such compensatory behavior involves derogatory treatment or devaluation of women (Willer et al., 2013).

Individuals displaying impersonal sexuality exhibit a notable inclination toward high sexual promiscuity coupled with a diminished commitment to sexual relationships. This tendency has also been referred to as unrestricted sociosexual orientation by Simpson and Gangestad (1991) and may be attributed to various factors, including a history of early abusive experiences and a proclivity toward general antisocial behaviors during adolescence (Malamuth, 2003). It is worth noting that both hostile masculinity (Anderson & Anderson, 2008; Carr & VanDeusen, 2004; Hall et al., 2006) and impersonal sexuality (Abrahams et al., 2004; Dunkle et al., 2006; Jewkes et al., 2006) have been empirically established as significant risk factors associated with sexual aggression.

The confluence model also underscores the importance of the interaction of these risk factors in the prediction of sexual aggression. In other words, the risk for sexual aggression increases overadditively for individuals with an impersonal sexuality and pronounced hostile masculinity. This interaction effect substantially enhances the explanatory power of the main effects of hostile masculinity and impersonal sexuality on sexual aggression, as evidenced by a considerable increase in the proportion of variance accounted for in sexual aggression (Abbey et al., 2006, 2011; Malamuth et al., 1991; Vega & Malamuth, 2007).

Thus, impersonal sexuality, hostile masculinity, and their interaction can be considered the core predictors of sexual aggression in the confluence model. Nevertheless, the model has always been inclusive for the integration of further risk factors. Particular attention, for example, has been paid to extensive alcohol (e.g., Smith & George, 2026) and pornography consumption (e.g., Baer et al., 2015). Additionally, less specific traits such as general aggression and hostility and other antisocial personality traits have been examined in the context of the confluence model (e.g., Knight & Sims‐Knight, 2003), which often overlap with the more “domain-specific” construct of hostile masculinity.

Another broad personality constellation, which might play a role in the explanation of sexual aggression, has been identified by Dean and Malamuth (1997), namely dominance/nurturance. This constellation describes two rather independent personality dimensions, which are known under different names (see Abele & Wojciske, 2018), such as masculinity and femininity (Bem, 1974), agency and communion (Abele & Wojciske, 2007; Bakan, 1966; Wiggins, 1997), instrumentality and expressiveness (Spence & Helmreich, 1980), or alpha and beta (Digman, 1997). Although there are nuances that differ between these conceptualizations of dominance and nurturance, a more specific description may be derived from the items in Bem’s (1974) Sex-Role Inventory (BSRI), which has been most commonly used to assess dominance and nurturance. There, dominant individuals are competitive, take risks, defend their own beliefs, are self-centered, act as leaders, and easily make decisions. Nurturant individuals, on the contrary, are characterized by the BSRI as warm, tender, affectionate, gentle, sympathetic, and sensitive to the needs of others. Dean and Malamuth’s (1997) findings revealed that in individuals characterized by a high dominance *and* low nurturance, the interaction between hostile masculinity and impersonal sexuality emerged as a robust predictor of sexual aggression. Conversely, this predictive power was notably absent in individuals exhibiting an orientation characterized by concern for others, exemplified by high nurturance and low dominance tendencies. This observation prompted Malamuth (1998) to expand the confluence model by incorporating the dimension of dominance/nurturance.

Given the content of the nurturance (femininity) scale in the BSRI, Dean and Malamuth (1997) proposed that the heightened empathy assumed for men with high nurturance and low dominance may account for their reduced susceptibility to sexual aggression compared to men with high dominance and low nurturance tendencies. This notion finds support in reports suggesting that individuals characterized by high nurturance and concurrently low dominance tend to exhibit greater levels of empathy than those with lower nurturance/higher dominance tendencies (Andrews et al., 2021; Karniol et al., 1998; Laurent & Hodges, 2009).

Furthermore, Wheeler et al. (2002) reported an intriguing moderating effect of empathy on the influence of hostile masculinity and impersonal sexuality on sexual aggression. More specifically, men characterized by low empathy, high levels of hostile masculinity, and a disposition toward impersonal rather than personal sexuality exhibited a significantly elevated risk of engaging in sexual aggression against women. In contrast, men displaying high empathy in conjunction with high hostile masculinity and high impersonal sexuality did not manifest a distinct risk profile for sexual aggression when compared to their counterparts who exhibited low hostile masculinity or low impersonal sexuality tendencies. More recently, the moderating influence of empathy on the relationship between hostile masculinity and impersonal sexuality on sexual aggression was also demonstrated by Hudson-Fledge et al. (2020) and, only for hostile masculinity, not impersonal sexuality by Malamuth et al. (2021).

These findings of empathy moderating the influence of hostile masculinity and impersonal sexuality on sexual aggression support Dean and Malamuth’s (1997) interpretation of their moderating effect of dominance versus nurturance on the relation between the risk factors and sexual aggression. Notably, in Wheeler et al. (2002) regression analysis, empathy did not exert a direct effect on sexual aggression; instead, its impact was solely discernible through its interaction with hostile masculinity and impersonal sexuality. This outcome aligns with a comprehensive meta-analysis based on 39 studies, which disclosed a mean correlation of only *r* = −.09 between empathy and sexual aggression (Vachon et al., 2014). These findings corroborate the hypothesis that the role of high dominance/low nurturance can be primarily understood through its overlap with empathy and its moderation of the effect of hostile masculinity and impersonal sexuality on sexual aggression in the confluence model (cf. Malamuth, 1998; Malamuth & Hald, 2017; Malamuth et al., 2021).

Nevertheless, an explicit and empirical examination of this postulate is currently lacking. Such an investigation is of particular significance, given that various other constructs, except for empathy, are intertwined with the traits of dominance and nurturance. For instance, anger and non-sexual aggression have been linked to high dominance and low nurturance tendencies (Milovchevich et al., 2001; Weisbuch et al., 1999). Thus, instead of (low) empathy, (high) anger may be deemed as a potential facilitator not only of men’s general aggression but also indirectly of their sexual aggression toward women (Anderson & Anderson, 2008). From this point of view, Dean and Malamuth (1997) question is still unanswered: “Are empathy skills per se the critical component attenuating the relationship between the risk factors and aggression, or is empathy merely one component of a larger personality dimension [i.e., dominance/nurturance] that may be necessary for the moderating effect to occur?” (p. 454).

The primary objective of the current study is to address this question empirically and to provide a more comprehensive understanding of the intricate relationship between sexual aggression and the dominance/nurturance dimension within the framework of the confluence model, with explicit consideration given to empathy. Proceeding from the above-outlined results, this investigation can have two different outcomes: On the one hand, it is plausible that empathy serves as the pivotal determinant within the dominance/nurturance dimension, thereby accounting for its association with sexual aggression or, more specifically, the moderating role in the confluence model. In such a scenario, the link between dominance/nurturance and sexual aggression should exhibit a decline or even become non-significant when controlled for empathy. On the other hand, the dominance/nurturance dimension represents a broader construct in contrast to empathy, encompassing an array of additional personality facets such as assertiveness, proclivity toward anger, and self-esteem. Moreover, owing to its direct connection to gender roles, dominance/nurturance may serve as a more robust indicator of vulnerability to gender-role stress. In this alternative scenario, dominance/nurturance may elucidate variance in sexual aggression (either uniquely or in interaction with hostile masculinity and impersonal sexuality) beyond the influence of empathy. The present study endeavors to disentangle these nuanced relationships to provide a deeper insight into the role of dominance/nurturance and empathy in the context of the confluence model on sexual aggression.

## Method

### Participants

The sample was part of a larger sample reported in detail in the study by Troche and Herzberg (2017). The empathy questionnaire was added to an online survey some weeks after starting data collection so that the empathy data of 300 men were available. Their age ranged from 18 to 65 years (M ± SD = 28.2 ± 9.5 years). While most men had high school graduation as the highest educational level (34.6%), 23.3% had a bachelor’s degree, and another 23.3% had a master’s degree. Another 13.4% had finished vocational school, and 5.3% had no higher education. Regarding relationship status, 41.7% indicated being single, and 42% were in a current relationship. Fifteen percent were married, and 1.3% were divorced or widowed. While 91% reported to be heterosexual, 9% were bisexual. Individuals who did not self-report being male and homosexual individuals were not included.

The online survey was advertised on social networks and Internet forums. Prior to their participation, the participants confirmed that they were 18 years of age or older. They were informed that the survey would present questions about sexuality and personality. The specific topic “sexual aggression” was not announced. We also informed participants that they could stop the survey at any time if, for example, they found the questions too intimate or uncomfortable. They were assured of complete anonymity during data collection and evaluation. After the last questions, participants were invited to write an email to the test administrator to take part in a prize draw to win one out of five Amazon vouchers worth 20€ and to be informed about the study results.

### Measures

*Sexual aggression*. For the assessment of sexual aggression, the German version of the Sexual Experience Survey (SES; Krahé et al., 1999) was used. The questionnaire consists of 12 items. The first two items do not refer to sexually aggressive behavior. Therefore, only the last ten items were used for computing a sexual aggression score, as recommended by Krahé et al. (1999). With these items, participants are asked whether they ever obtained sexual intercourse or other sexual activities (kissing, petting, oral, or anal intercourse) by verbally manipulating or physically forcing a woman or by decreasing her resistance by giving her alcohol or other drugs. The count score of positive responses can range from 0 to 10. According to Krahé et al. (1999), the average percentage of stability with a test–retest interval of three to four weeks is 95.6%.

*Hostile masculinity*. Hostile masculinity was measured using the Hostile Sexism Scale of the Ambivalent Sexism Inventory (ASI; Eckes & Six-Materna, 1999). The 11 items of the Hostile Sexism Scale of the ASI are responded to on a six-point rating scale (“totally disagree” to “totally agree”) with reliability ranging from .78 to .87 (Eckes & Six-Materna, 1999). An example is “Women are too easily offended” (Glick & Fiske, 1996, p. 512). Items were recoded when necessary, and the test score was computed as the mean score across all hostile sexism items. The Hostile Sexism Scale captures core features such as hostility, distrust, and antagonism toward women (Eckes & Six-Materna, 1999) and, as might be expected, was found to be related to narcissism (e.g., Grubbs et al., 2014; Keiller, 2010). Thus, it can be assumed that the Hostile Sexism Scale is a valid representation of the first component of Malamuth and Hald’s conceptualization of hostile masculinity. It should be noted, however, that other components of the hostile masculinity construct, such as sexual dominance, adversarial sexual beliefs, or rape myth acceptance, were not considered. Although the AMMSA (Modern Myths about Sexual Aggression Questionnaire; Gerger et al. 2007) data were available as an additional measure of hostile masculinity, we decided not to include it in the present analyses because it was strongly correlated with the Hostile Sexism Scale (*r* = .75, corrected *r* = .82), and both predicted sexual aggression to a similar degree (*r* = .29 vs. .30). Furthermore, we decided to use only the Hostile Sexism Scale out of the two hostile masculinity measures to minimize the risk of multicollinearity in the count regression models (see below).

*Impersonal vs. personal sexuality*. As a measure of impersonal sexuality, we used the revised Sociosexual Orientation Inventory (SOI-R; Penke & Asendorpf, 2008). It assesses sociosexual behavior, attitudes, and desire. The SOI-R contains three items on sociosexual behavior (e.g., “With how many different partners have you had sexual intercourse on one and only one occasion?”), three on sociosexual attitudes (e.g., “Sex without love is ok”), and three items on sociosexual desire (e.g., “In everyday life, how often do you have spontaneous fantasies about having sex with someone you have just met?”). All items were answered on a 9-point rating scale. Penke and Asendorpf (2008) reported Cronbach’s alpha of .83 for the total score across the nine items.

*Dominance vs. nurturance*. The revised German version of the Bem Sex-Role Inventory (BSRI-R; Troche & Rammsayer, 2011) was used to measure dominance (masculinity) and nurturance (femininity) with 15 items each. The internal consistencies of the two scales were *α* = .86 in the study by Troche and Rammsayer (2011). The participants responded to the items on a seven-point Likert scale ranging from “never or almost never true” to “always true.” Separate mean scores of the responses were computed for dominance and nurturance. Afterward, the mean score on the nurturance scale was subtracted from the mean score on the dominance scale (cf. Dean & Malamuth, 1997). Thus, high positive scores indicate high dominance and, concurrently, low nurturance, while high negative scores indicate high nurturance but low dominance. However, we acknowledge that the latent correlation between dominance and nurturance in our sample was low (*r* = .09, *p* = .153), suggesting that the constructs may not form a unidimensional continuum. Although we initially followed Dean and Malamuth’s (1997) approach of creating a difference score, we conducted supplementary analyses entering dominance and nurturance as separate predictors and moderators. These additional models revealed that neither dominance nor nurturance significantly moderated the effects of hostile masculinity or impersonal sexuality on sexual aggression. This supports the conclusion that the subtraction approach did not distort the findings and that neither construct—considered separately—explained meaningful additional variance beyond empathy.

*Empathy*. The German version of Davis’ (1983) Interpersonal Reactivity Index (IRI), adapted and translated by Paulus (2009), was used to measure perspective taking, fantasy, empathic concern, and personal distress as four aspects of empathy with four items each. Each item was answered on a five-point Likert scale (“strongly disagree” to “strongly agree”). Paulus (2009) reported split-half reliability of *r* = .80 for the total test score. Example items are “Before I criticize someone, I try to imagine how things look from his/her perspective.” for perspective taking, “Watching a good movie, I can put myself easily in the main character’s position.” for fantasy, “I have warm feelings for people who are not so well.” for empathic concern, and “In emergency situations I feel anxiously and uncomfortably.” for emotional distress. A mean score across all 16 items was computed for each individual, with higher scores indicating high empathy.

### Statistical Analyses

We tested our hypotheses with three latent regression models. Since the dependent variable SES was a count variable, we used count regression models (Poisson regression, zero-inflated Poisson model, and negative binomial model) of count outcomes.^1^ We compared the different models to determine the best-fitting model. Using latent count regression models was necessary because we modeled the dominance/nurturance dimensions as a difference score to capture the individual standing on the dominance/nurturance continuum. Since difference scores have low reliability (overall & Woodward, 1975), we circumvent the low-reliability problem using a latent difference score approach (McArdle, 2001). The latent factors were formed from the items of the respective scales.

Three nested models were tested, all containing “impersonal sexuality,” “hostile masculinity,” “dominance/nurturance,” and “empathy” as latent variables (and their interactions) as well as “sexual aggression” as the dependent variable. However, the influence of empathy on sexual aggression was constrained to zero in the first model, and the influence of dominance/nurturance on sexual aggression was constrained to zero in the second model. Both influences were freely estimated concurrently in the third model. More specifically, Model 1 included the following latent variables: the latent difference variable obtained from the dominance and nurturance scales of the BSRI-R, a latent variable “impersonal sexuality” derived from the nine SOI-R items, and a latent variable “hostile masculinity” derived from the eleven ASI items. Additionally, we examined three two-way interactions: (1) between dominance/nurturance and impersonal sexuality, (2) between dominance/nurturance and hostile masculinity, and (3) between impersonal sexuality and hostile masculinity. In addition to these two-way interactions, we also explored a three-way latent interaction between dominance/nurturance, impersonal sexuality, and hostile masculinity. This was explored to understand how the interaction between impersonal sexuality and hostile masculinity may be moderated by levels of dominance/nurturance.

**Fig. 1:**
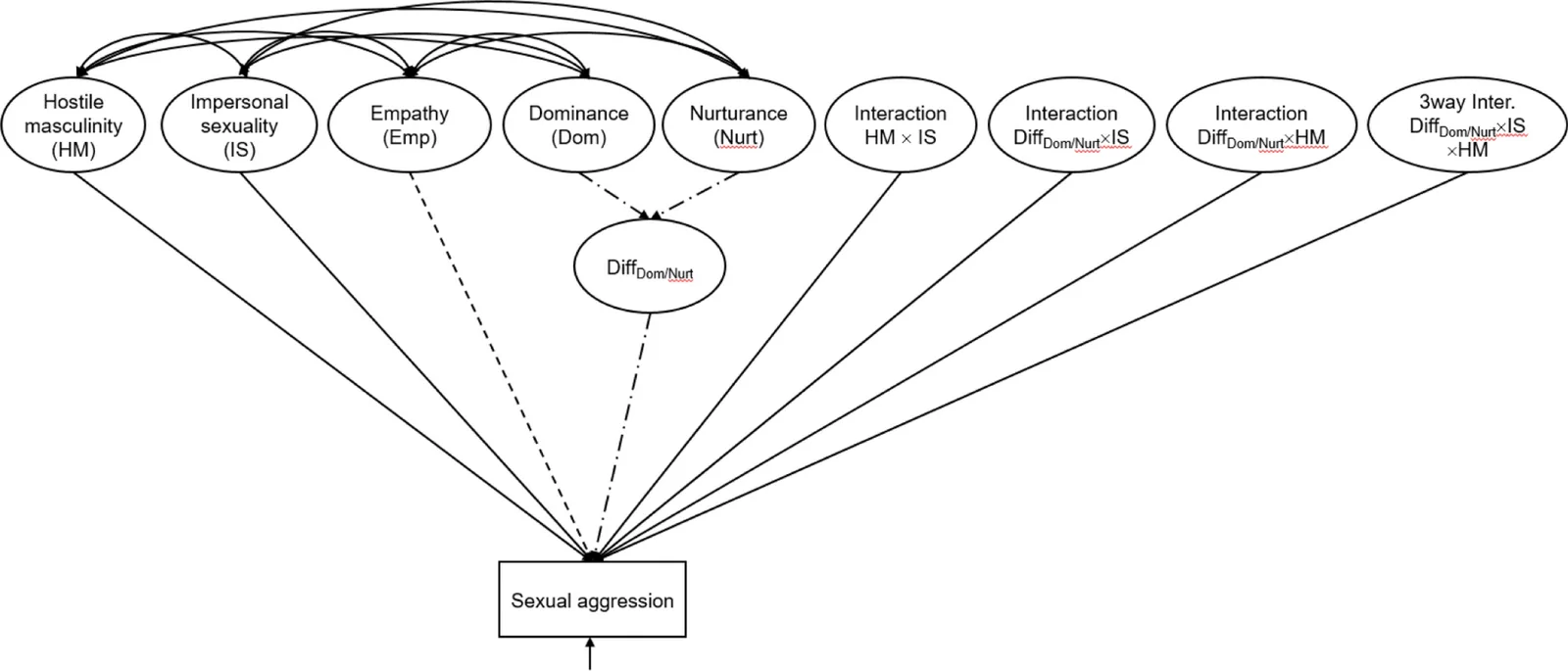
The negative binomial regression model Note: Depicted are all latent variables, and the two-way and three-way latent interactions. Model 3 contains all depicted variables. In Model 2, the latent difference variable is omitted, indicated by the dash-dotted line. In Model 1, the latent empathy variable is omitted, indicated by the dashed line

Model 2 was the same as Model 1, except that the latent difference variable was replaced by a latent empathy variable extracted from the 16 IRI items. Finally, Model 3 contained impersonal sexuality, hostile masculinity, the latent difference variable, the latent empathy variable, and the two-way and three-way latent interactions. Figure 1 depicts the models.

According to Heeringa et al. (2020), the first step is the specification stage, where the appropriate regression model that accounts for potential overdispersion is selected. The second step is the estimation stage. Since our models include a difference score and interaction terms, we used count regression models based on structural equation modeling. These models allow for the specification of latent interactions and difference scores, accounting for measurement error (Kiefer et al., 2024). Cameron and Trivedi (2013) recommended computing robust standard errors when analyzing count models. Thus, we used the restricted maximum likelihood (MLR) estimator, which computes robust standard errors.

## Results

Means, standard deviations, and internal consistencies of all scale sum scores and their intercorrelations are presented in Table 1. Intercorrelations were mostly low except for the correlations between empathy and nurturance, *r* = .48, *p* < .001, as well as the correlation between empathy and the dominance/nurturance difference scores,* r* = −.52, *p* < .001. Particularly important for the present purpose, sexual aggression was significantly positively associated with impersonal sexuality and hostile masculinity, dominance, dominance/nurturance, but not with nurturance. Furthermore, sexual aggression correlated significantly negatively with empathy.

**Table 1:** Mean, standard deviation, and range of as well as intercorrelations among age, dominance, nurturance, perspective taking, empathic concern, global empathy, and sexual aggression

		M	SD	α	ω	Range	1	2	3	4	5	6
1	Age	28.15	9.48	–		18.0–65.0	–					
2	BSRI-R instrumentality (dominance)	4.52	.90	.90	.92	1.00–7.00	.01	–				
3	BSRI-R expressivity (nurturance)	4.63	.90	.89	.92	1.00–6.53	− .03	.23	–			
4	Difference dominance/nurturance					− 2.40–4.67						
5	Sociosexual orientation	4.89	1.75	.85	.86	1.22–9.22	.13	.22	.01	–		
6	Hostile masculinity	3.57	1.03	.89	.90	1.00–6.00	− .08	.29	− .05	.18	–	
7	Empathy	3.56	.65	.80	.86	1.00–5.00	.04	− .05	.50	− .10	− .11	–
8	SES	0.72	1.26	.96^a^	.98^a^	1–9	.02	− .07	.08	− .18	− .21	.21

$$\left| r \right|$$> .20* p* < .001., $$\left| r \right|$$> .12* p* < .05.. N = 300.

^a^Reliability based on polychoric correlations. ω = McDonald’s omega. BSRI-R = revised version of the Bem Sex-Role Inventory; SES = Sexual Experience Survey.

BSRI-R dominance and nurturance scores correlated only slightly but significantly with each other, *r* = .23, *p* < .01. The latent correlation between dominance and nurturance was *r* = .09, *p* = .153, with a 95% CI ranging from -.01 to .20.

Reliabilities (Cronbach’s alpha and McDonald’s omega) were good for all scales (see Table 1). Since sexual aggression measured with the Sexual Experience Survey is a count variable, we based the reliability estimates on polychoric correlations. Both measures indicated excellent reliability of the SES scale.

**Fig. 2:**
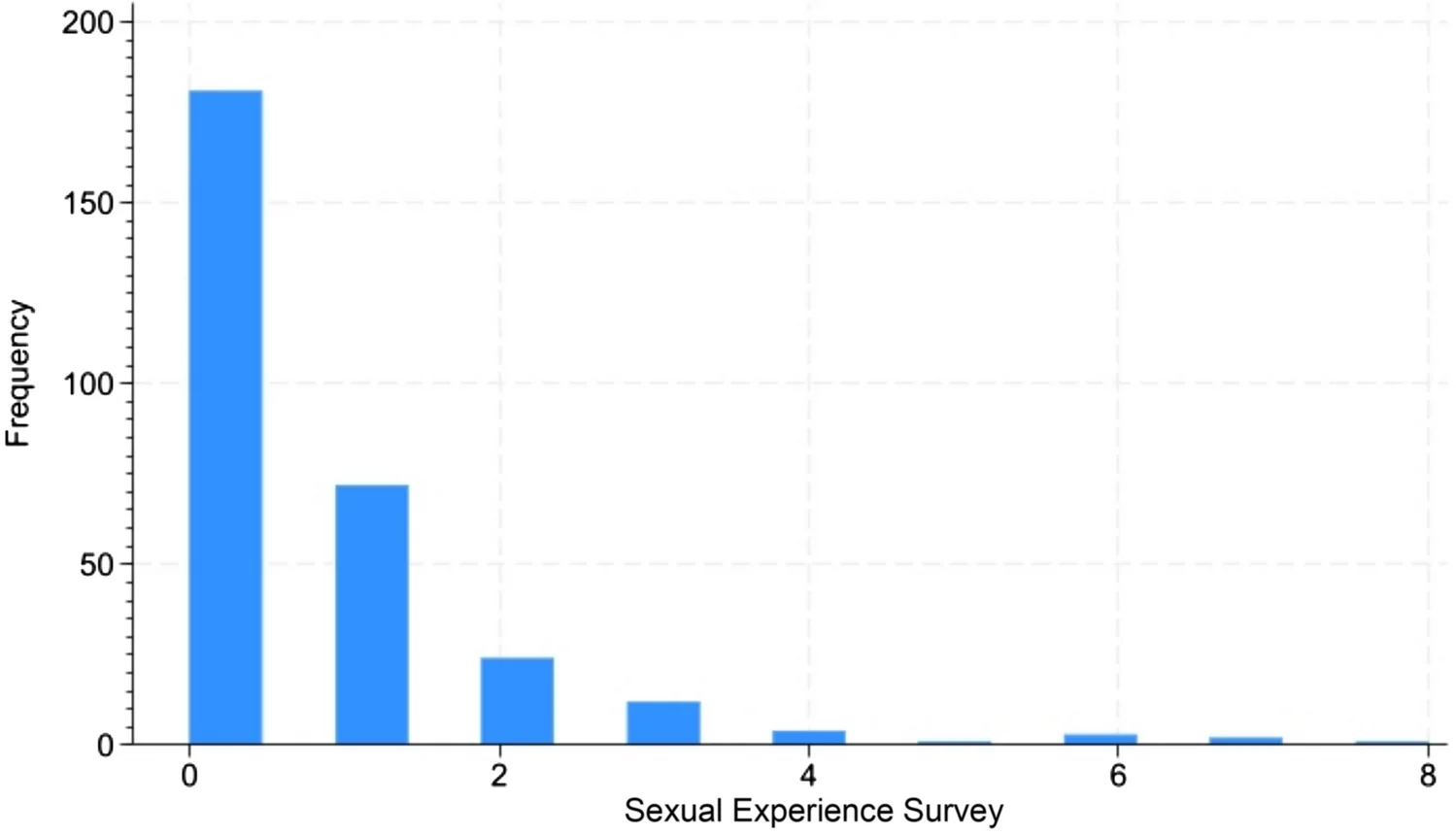
Counts of sexual aggression

The count proportion of zeros is 0.603, and the maximal value of sexual aggression counts is 8 (see Fig. 2). As shown in Table 1, the standard deviation of the SES scores exceeds their mean value, which points to overdispersion. As recommended for such a case (e.g., Coxe et al., 2009), we first fitted a Poisson regression model and tested for overdispersion. This test was significant, *z* = 3.03, *p* = .0012, indicating overdispersion. Thus, a negative binomial regression model was computed. The interpretation of the dispersion parameter differs for the negative binomial model; a significant parameter indicates that the Poisson model is inferior. The dispersion parameter for the negative binomial model was statistically significant (*α* = 0.64, *p* = .004). The next step was to test for zero inflation of the count variable. The test for zero inflation in the negative binomial model fit was not significant, *p* = .562, and the ratio (1.01) of observed and predicted zeros was within the tolerance range (tolerance = 0.05). This means an absence of zero inflation under the fitted model. Thus, the negative binomial model was sufficient and there was no need for an inflation part, respectively, zero-inflated regression models. A final check of the negative binomial model’s appropriateness was to prove whether the residuals were normally distributed. The Q-Q plot (supplement Fig. 1) shows that the residuals followed a diagonal line. Furthermore, the Kolmogorov–Smirnov statistic was not statistically significant, *p* = .487. Thus, the residuals were normally distributed, and a negative binomial model was more appropriate than the Poisson model.

We computed three negative binomial models and compared the model fit (see Table 2). To provide a comprehensive comparison, parameter estimates, standard errors, and *p* values for all predictors and interactions in Models 1, 2, and 3 are presented in Table 3.^2^ In Model 1, where the effects of empathy were constrained to zero, only hostile masculinity (b = 0.435, *p* = .024) and impersonal sexuality (b = 0.410, *p* = .022) were significant positive predictors of sexual aggression, but the latent difference variable obtained from the dominance and nurturance scales was not (b = 0.118, *p* = .410), and no interaction was significant (all *p*s > .25). Thus, the significant correlational relationship between dominance/nurturance and sexual aggression at the manifest level (see Table 1) could not be corroborated at the latent level when dominance/nurturance was examined as a predictor of sexual aggression together with impersonal sexuality and hostile masculinity.

**Table 2:** Model fit for three latent regression models with sexual aggression as count outcome

Regression model	Model	Parameters	LL	AIC	BIC	aBic	AICC
Negative binomial	1	193	− 28,525.46	57,436.92	58,151.75	57,539.67	58,143.37
	2	193	− 28,515.65	57,417.30	58,132.13	57,520.04	58,123.75
	3	197	− 28,514.58	57,423.15	58,152.80	57,528.03	58,187.98

Model 1 without empathy, Model 2: without latent difference score, Model 3: with latent difference score and empathy

**Table 3:** Parameter estimates for three latent variable models

Predictor	Parameters			SE			*p*		
	Model 1	Model 2	Model 3	Model 1	Model 2	Model 3	Model 1	Model 2	Model 3
Intercept sexual aggression	− .520	− .647	− .683	.154	.134	.137	.001	< .001	< .001
Hostile masculinity	.435	.604	.637	.193	.128	.128	.024	< .001	< .001
Impersonal sexuality	.410	.490	.495	.179	.157	.153	.022	.002	.001
Dominance vs. nurturance (a)	.118	–	− .124	.143	–	.129	.410	–	.335
Empathy (b)	–	− .703	− .946	.274	.274	.355	.010	.010	.008
Hostile masculinity x a or b	− .090^a^	.309^b^	.021^a^ .354^b^	.150	.214	.217^a^ .110^b^	.548	.147	.849^a^ .103^b^
Impersonal sexuality x a or b	− .093^a^	.574	.040^a^ .613^b^	.111	.338	.456^a^ .145^b^	.402	.089	.179^a^ .784^b^
Hostile masculinity x Impersonal sexuality	− .102	− .196	− .248	.199	.131	.136	.609	.134	.068
Hostile masculinity x Impersonal sexuality x a or b	.107^a^	− .348^b^	.081^a^-.117^b^	.095	.189	.109^a^ .267^b^	.258	.065	.459^a^ .660^b^

CI = confidence interval; LL = lower limit; UL = upper limit

In Model 2, where the effects of dominance/nurturance were restricted to zero, hostile masculinity (b = 0.604, *p* < .001) and impersonal sexuality (b = 0.490, *p* = .002) remained significant positive predictors. Empathy was a significant negative predictor (b = −0.703, *p* = .010); interactions were non-significant (*p*s > .05, though the three-way interaction just failed to reach statistical significance, *p* = .065).

In Model 3, where the effects of both the latent dominance/nurturance variable and empathy were freely estimated, hostile masculinity (b = 0.637, *p* < .001) and impersonal sexuality (b = 0.495, *p* = .001) again predicted sexual aggression. Empathy remained a significant negative predictor (b = −0.946, *p* = .008), but the latent difference variable obtained from the dominance and nurturance scales was not (b = −0.124, *p* = .335), with no significant interactions (*p*s > .05).

As can be taken from Table 2, Model 2 (without the latent difference variable from the dominance and nurturance scales but with the latent empathy variable) provided the best model/data fit according to AIC, BIC, and sample-size-adjusted BIC. These results across models indicated that empathy accounted for unique variance in sexual aggression beyond the latent difference variable obtained from the dominance and nurturance scales. However, since dominance/nurturance did not exert an effect on sexual aggression at all, this effect could not be replaced or explained by empathy.^3^

The non-significant interactions have the logical consequence that Model 2 can be simplified by retaining only the paths that have become significant in the latent model. Furthermore, the excluded difference score in Model 2 makes it sufficient to compute the negative binomial model with manifest scales, facilitating the interpretation of the model. The parameter estimates are given in Table 4. Holding all other variables constant, a one-unit increase in hostile masculinity or impersonal sexuality would multiply the expected rate at which sexual aggression occurred by a factor of 1.46 and 1.25, respectively (see penultimate column). Every one-unit increase in the value of empathy decreased the expected rate at which sexual aggression occurred by .66 times, provided that other predictor variables were constant. For ease of interpretation, we computed the percentage change in expected count (last column Table 4) and visualized the effects (supplement Fig. 2).

**Table 4:** Parameter estimates for the purified Model 2 (manifest variable model)

Predictor	Parameters	SE	95% CI		*p*	e^(Parameter)^	%
			LL	UL			
Intercept sexual aggression	− 1.49	.89	− 3.23	.25	.094		
Hostile masculinity	.38	.10	.18	.57	< .001	1.46	45.8
Impersonal sexuality	.22	.06	.10	.33	< .001	1.25	24.6
Empathy	− .42	.17	− .76	− .08	.014	0.66	− 34.4

CI = confidence interval; *LL* = lower limit; *UL* = upper limit. % = percentage change in expected count

**Fig. 3:**
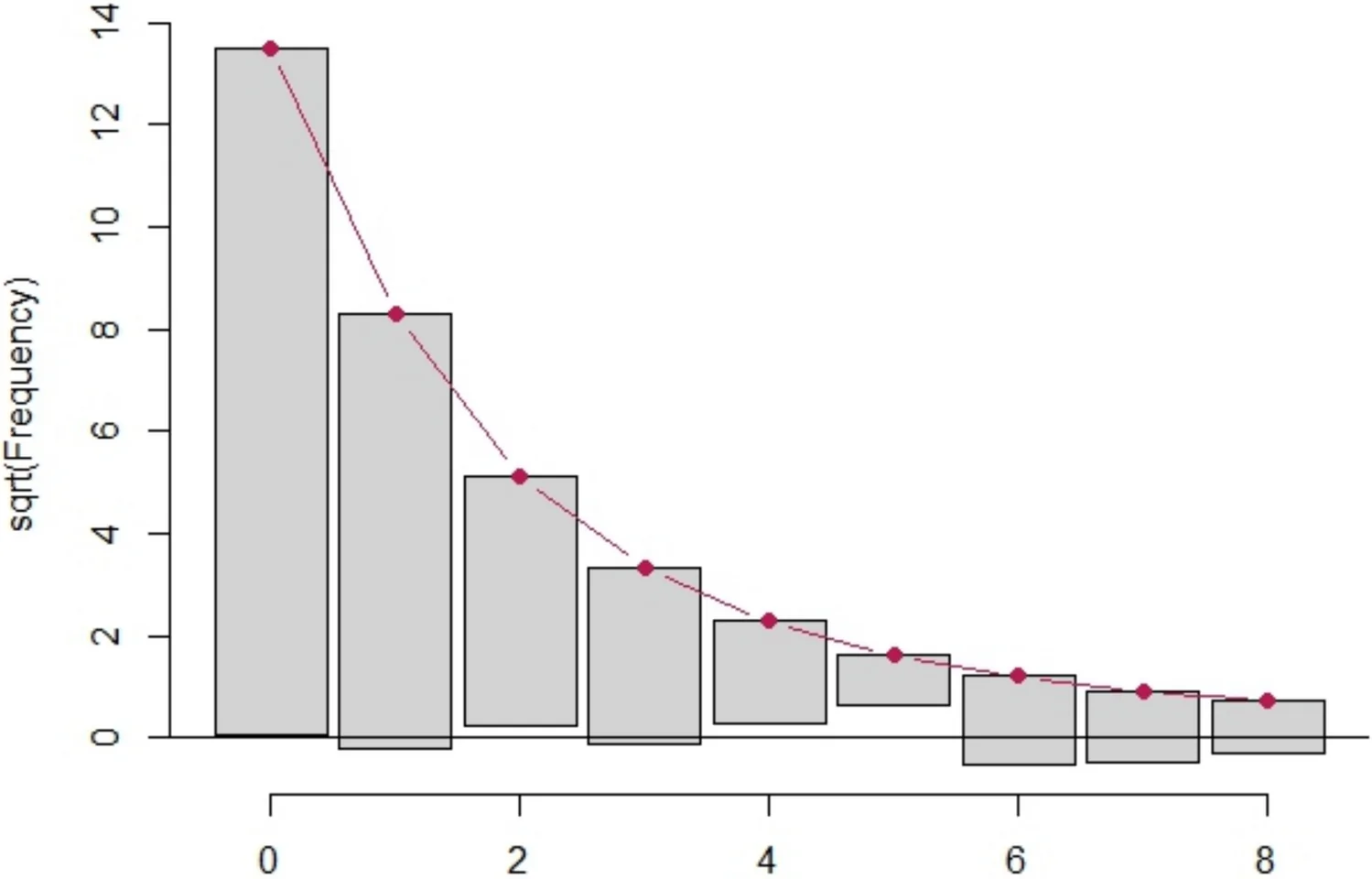
Expected and observed counts from Model 2

The divergence-based R^2^ (Kullback–Leibler) was .21. We plotted the residuals vs. predicted values to further assess the adequacy of Model 2. As indicated by Fig. 3 in the supplement, there was neither fanning nor uneven spreading of residuals across fitted values nor curvilinearity, indicating missing higher-order terms or an inappropriate link function. Thus, Model 2 was able to fit the observed counts with the predictors used. The accuracy of the model was further supported by plotting the observed and expected counts as given in Fig. 3. Expected counts are shown by the thick line; observed counts are shown as bars hanging from the line of expected counts. Interpretation is as follows: If a bar does not reach the zero line, then the model overpredicts a particular count bin, and if the bar exceeds the zero line, it under-predicts. As can be seen in Fig. 3, there was a generally good agreement between the expected and observed counts, with a small amount of under-prediction of counts six, seven, and eight and a small amount of overprediction of counts four and five. The predicted probabilities for counts in Model 2 are given in Table 5.

**Table 5:** Predicted probabilities for counts for Model 2

	Counts								
	0	1	2	3	4	5	6	7	8
Model 2	.621	.252	.087	.028	.009	.003	.001	< .001	< .001

**Fig. 4:**
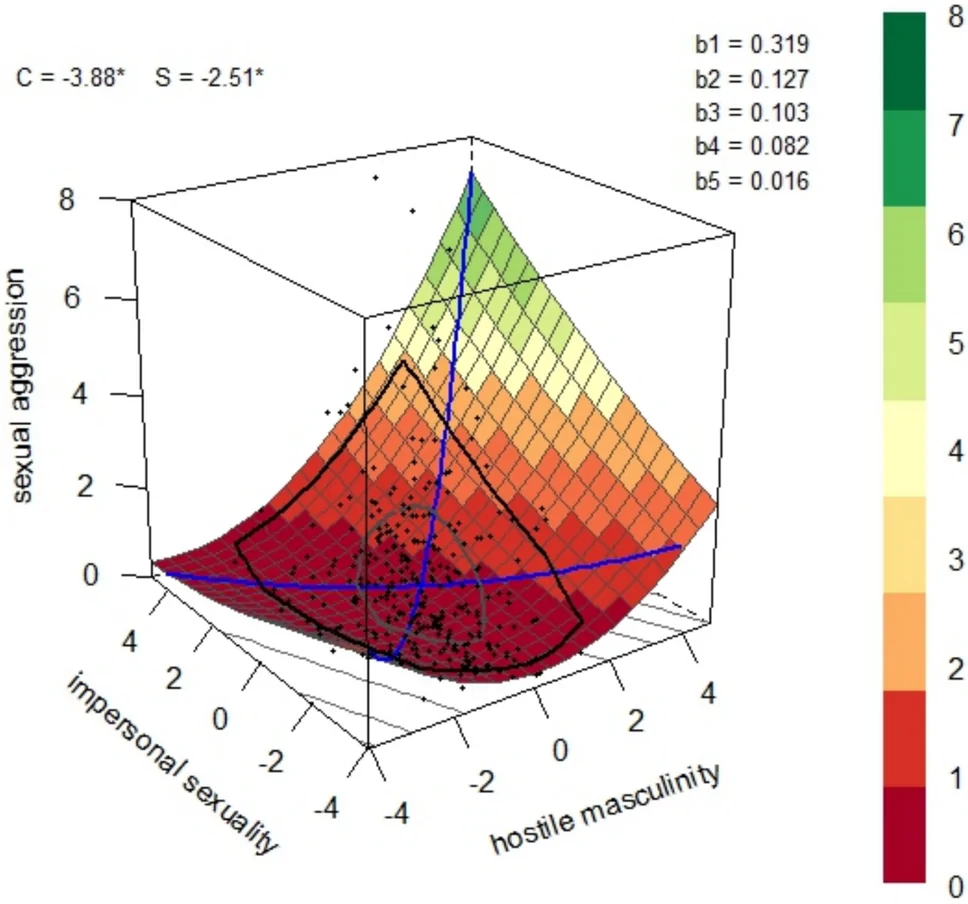
Response surface plot for the SRSQD model

To further explore the interplay of hostile masculinity and impersonal sexuality on sexual aggression, we conducted a series of response surface analyses (RSA) using the RSA package (version 0.10.6) in R.^4^ The full polynomial model was compared with a range of theoretically structured models. The shifted and rotated squared difference model (SRSQD) showed superior fit, as indicated by the lowest corrected Akaike information criterion (AICc = 938.62) and excellent model fit indices (CFI = 1.000, SRMR = 0.011, R^2^ = .189). The SRSQD model retained statistically significant main effects and key interaction terms. Hostile masculinity showed a strong positive effect on sexual aggression (b = 0.319, *p* < .001), consistent with prior findings that frame hostile masculinity as a central risk factor. Impersonal sexuality also predicted increased sexual aggression (b = 0.127, *p* = .001), albeit to a lesser extent. A significant interaction between hostile masculinity and impersonal sexuality (b = 0.082, *p* = .011) suggests a synergistic effect, whereby the combination of high hostile masculinity and impersonal sexuality further amplifies sexual aggression risk (see Fig. 4). Polynomial terms indicated a significant quadratic effect for hostile masculinity (b = 0.103, *p* = .024), highlighting a nonlinear increase in aggression at higher hostility levels. The quadratic term for impersonal sexuality was not significant (*p* = .133), suggesting linearity in its association. Surface tests revealed significant curvature along the line of congruence (a_2_ = 0.202, *p* = .006) and a shifted ridge (a_3_ = 0.192, *p* = .024), implying that sexual aggression is not solely a function of equal levels of hostile masculinity and impersonal sexuality, but particularly elevated when hostile masculinity is high even if impersonal sexuality is moderate or low. The stationary point, located at hostile masculinity = 0.726 and impersonal sexuality = −5.706, lies away from the line of congruence, further supporting the presence of asymmetrical effects. The first principal axis was laterally shifted from the LOC (C1 = 3.466), reinforcing the interpretation that extreme levels of hostile masculinity drive the model’s dynamics.

## Discussion

The present study builds upon the confluence model of sexual aggression (Malamuth, 1998; Malamuth & Hard, 2016; Malamuth et al., 2021), comparing the roles of dominance/nurturance and empathy within this framework. We tested three models to determine which variables and interactions best explained the occurrence of sexually aggressive behaviors among men. In all three models, hostile masculinity and impersonal sexuality were systematically related to sexual aggression, underscoring their role as core predictors of sexual aggression in the confluence model. Model 1 revealed that dominance/nurturance was not substantially related to sexual aggression—neither directly nor in interaction with impersonal sexuality and hostile masculinity. Model 2, which included empathy but suppressed the potential influence of dominance/nurturance on sexual aggression, revealed a significant and negative main effect of empathy on sexual aggression. Since dominance/nurturance was not significantly related to sexual aggression, there was no effect that could be explained by empathy, despite the relatively high correlation between empathy and dominance/nurturance. The exclusion of dominance/nurturance, therefore, did not attenuate the confluence model’s explanatory power so that Model 3 did not explain the data better than Model 2. In sum, the best prediction of sexual aggression was achieved with impersonal sexuality, hostile masculinity, and empathy, while dominance/nurturance did not improve the prediction of sexual aggression beyond the other variables.

### The Core Risk Factors in the Confluence Model: Hostile Masculinity and Impersonal Sexuality

Consistent with the confluence model, both hostile masculinity and impersonal sexuality were confirmed as significant predictors of sexual aggression. Men who scored higher on measures of hostile masculinity and impersonal sexuality reported higher rates of sexually aggressive behaviors. These findings corroborate previous studies (e.g., Abbey et al., 2011; Malamuth et al., 1991) that identified these features as core risk factors for sexual aggression.

In contrast to our expectations, however, the interaction between impersonal sexuality and hostile masculinity did not reach statistical significance. A possible reason might be seen in the fact that we focused on only one component of hostile masculinity (i.e., hostile and distrustful orientation toward women) but neglected other components (e.g., sexual dominance). We cannot rule out that, with a broader operationalization of hostile masculinity, the expected interaction effect would have emerged. Similarly, the three-way interaction in Model 2 between impersonal sexuality, hostile masculinity, and empathy on sexual aggression just failed to reach statistical significance, maybe because the sample size was not large enough. On the other hand, in a previous study with two non-heterosexual samples, we also failed to observe this interaction effect, but only when we switched from the ordinary regression to Poisson regression (Waldis et al., 2023). Notably, most studies reporting interactions within the confluence model have not used count regression models. Count regression models, such as Poisson or negative binomial regression, use a log link function, introducing nonlinearity between the predictors and the outcome. This nonlinearity can make detecting interactions more challenging compared to linear regression, as the interaction term in a log-linear model affects the outcome multiplicatively rather than additively. At the same time, it is also possible that interaction effects reported in previous research using ordinary least squares regression may have been overestimated due to violations of distributional assumptions inherent to count data. Therefore, the absence of a significant interaction effect between hostile masculinity and impersonal sexuality in our study may not necessarily indicate a failure to detect an existing effect but rather reflect a more conservative and accurate estimation using appropriate count regression techniques.

The additional response surface analyses, however, indicate that this conclusion from the negative binomial models might be premature, as the additional analyses uncovered the interaction effect from impersonal sexuality and hostile masculinity on sexual aggression. This finding corroborates the claim that hostile masculinity and impersonal sexuality are mutually reinforcing pathways to sexual aggression (Bruera et al., 2023; Malamuth, 1998, 2003; Malamuth & Hald, 2017). The RSA findings extend this idea by specifying the nature of their interaction: While both variables contribute independently, their combination—especially high Host with any level of SOI—markedly elevates risk. This suggests that the interaction is not symmetrical but is critically driven by hostile masculinity. Therefore, the RSA findings do not contradict the negative binomial results but rather elaborate upon them, specifying the precise, asymmetric nature of the interaction that a standard regression coefficient alone could not fully illuminate. Our study thus underscores the value of employing complementary analytical techniques to fully unravel the complex, synergistic pathways to sexual aggression.

### The Roles of Dominance/Nurturance and Empathy in the Confluence Model

The comparison of the three binomial models demonstrates that the latent difference variable obtained from the dominance and nurturance scales does not exhibit independent main or moderating effects, supporting the conclusion that its prior role in the confluence model is likely attributable to its shared variance with empathy (*r* = −0.52).

In accordance with previous studies (Andrews et al., 2021; Karniol et al., 1998; Laurent & Hodges, 2009), we observed a moderately strong positive correlation between empathy and nurturance and a weak negative correlation between empathy and dominance, which resulted in a moderately strong negative correlation between empathy and dominance/nurturance. Previous studies have emphasized that empathy is part of the broad personality dimension nurturance (e.g., Abele & Wojciszke, 2007) so that the present correlation is likely to reflect the conceptual overlap between nurturance and empathy. It was this conceptual overlap that made Dean and Malamuth (1997) assume that the moderating effect of dominance/nurturance on the relationship between risk factors and sexual aggression might be explained in terms of empathy. In the present study, however, we could not replicate Dean and Malamuth’s result. Dominance/nurturance correlated with sexual aggression at the manifest level. At the latent level and embedded in a regression model together with hostile masculinity and impersonal sexuality, dominance/nurturance did not contribute to the explanation of variance in sexual aggression (cf. Model 1). This was true for the direct effects as well as the interaction effects. This rendered the question redundant regarding whether empathy could replace the role of dominance/nurturance in the confluence model as there was no substantial effect to replace. Tentatively, this result suggests that dominance/nurturance should no longer be considered in studies on the confluence model. This is also advisable from a methodological point of view since the computation of a difference score from two widely independent scores of dominance and nurturance (or masculinity and femininity) has already been criticized shortly after Bem (1974) introduced this measure (Pedhazur & Tetenbaum, 1979; Spence et al., 1975).

In contrast to dominance/nurturance, our findings indicated that empathy served as a protective factor against sexual aggression. Higher levels of empathy were associated with a lower likelihood of engaging in sexually aggressive behaviors. This was true at the manifest level (see Table 1) and the latent level (Model 2) and supports previous research (Hudson-Fledge et al., 2020; Malamuth et al., 2021; Wheeler et al., 2002). Most of the previous research, however, emphasized the moderating role of empathy in the relationship between hostile masculinity, impersonal sexuality, and sexual aggression. In our negative binomial regression model, however, empathy had a main effect on sexual aggression, but the three-way interaction with impersonal sexuality and hostile masculinity (just) failed to reach significance, not corroborating a moderating role of empathy. Here, the same arguments can be put forward as above for the lack of interaction between impersonal sexuality and hostile masculinity. If we take the results as they are, high empathy reduces the likelihood of actually engaging in sexually aggressive behavior even in the presence of other risk factors for sexual aggression. In other words, when a man can take the perspective of a potential victim of their sexually aggressive behavior and can imagine how this behavior would feel for the victim, it is less likely that the behavior is carried out. This tendency is independent of his hostility against women and his tendency for impersonal sexuality (for a more nuanced consideration of possible mechanisms by which empathy deficits facilitate sexual aggression, see Barnett & Mann, 2013).

Finally, we address a reviewer’s insightful query regarding the prevalence and theoretical rationale for a profile characterized by high hostile masculinity, high impersonal sexuality, and high empathy. Theoretically, such a combination is uncommon, as hostile masculinity and impersonal sexuality are typically associated with delinquent behaviors, and empathy deficits (Marshall et al., 1995; Wheeler et al., 2002). Developmental pathways suggest that men high in hostile masculinity and impersonal sexuality often exhibit reduced empathic abilities due to adversarial interpersonal schemas and objectifying attitudes toward sex, making high empathy in this context atypical (Malamuth et al., 2021). Empirical correlations support this: Hostile masculinity and impersonal sexuality tend to correlate positively with each other (*r* ≈ .30–.50) and negatively with empathy (*r* ≈ − .20–.40 across studies; e.g., Abbey et al., 2007; Parkhill & Abbey, 2008). To quantify prevalence, we dichotomized each scale using an upper-tertile split to define high scores (hostile masculinity ≥ 4.0, impersonal sexuality ≥ 6.0, empathy ≥ 3.7) and assigned participants to profiles based on the conjunction of these categorical indicators. The risk profile of high hostile masculinity, high impersonal sexuality, and low empathy—aligned with the confluence model’s high-risk constellation—was evident in only 5.7% of our sample, suggesting a distinct subgroup prone to sexual aggression (see also Troche & Herzberg, 2017, for a similar person-centered approach identifying elevated aggression in low nurturance profiles). Conversely, the queried profile (high hostile masculinity, high impersonal sexuality, high empathy) was even rarer, appearing in < 1% of participants, underscoring its improbability. This low prevalence of the core risk combination likely contributed to the non-significant three-way interaction in our model, as statistical power to detect moderating effects diminishes when pivotal subgroups are small (Aiken & West, 1991). In sum, our results suggest that future studies on the confluence model are well advised to incorporate measures of empathy (instead of dominance/nurturance) to consider its attenuating influence on sexual aggression.

### Implications for Prevention and Intervention

Research on sexual aggression has made great progress in identifying important risk factors, which should support the development of effective prevention and intervention programs. Nevertheless, to date, studies showing the effectiveness of prevention programs are relatively scarce (e.g., Knight & Sims-Knight, 2009; Newlands & O’Donohue, 2016). Programs on enabling bystanders to intervene in sexually violent situations have been shown to be promising (Bell et al., 2019). However, despite the increasing knowledge about risk factors of sexual aggression, it remains challenging to change relevant attitudes and sexually aggressive behavior in men with strong expressions of these risk factors. Knight and Sims-Knight (2009) argued that programs focusing on the attitudes related to sexual aggression (such as hostility against women) are not successful over time, as they do not change the broad and highly stable traits underlying these attitudes. Malamuth et al. (2018) even warned that some programs could do more harm than good since boomerang effects were caused, especially in high-risk men, i.e., some programs resulted in higher rather than lower sexual aggressive tendencies. This might be caused by reactance to the intervention, when it is experienced as restricting their free thinking, feeling, and behaving.

In their overview, Malamuth et al. (2018) cited two earlier studies that reported successful interventions targeting risk factors in high-risk men (Schewe & O’Donohue 1993, 1996). These authors developed programs to influence rape supportive cognitions and victim empathy, which were partially successful in increasing empathy with the victims and decreasing some risk factors, such as attraction to sexual aggression and acceptance of interpersonal violence. Thus, prevention programs targeting empathy with victims may be successful.

In this context, however, the question of whether empathy interacts with impersonal sexuality and hostile masculinity in the explanation of sexual aggression becomes crucial. If such an interaction actually exists (as suggested by Hudson-Fledge et al., 2020; Malamuth et al., 2021; Wheeler et al., 2002) and the present study was just unable to detect it, a target group or profile could be assumed for which intervention programs should be designed. An increase in empathy should, in this case, lower the risk for men in this group to act sexually aggressively. If the interaction effect, however, is an artificial consequence of linear regression analyses, the present results would mean that there are (at least) two groups of men committing sexual aggression: some for reasons of low empathy and others due to their hostile masculinity or impersonal sexuality. An intervention program focusing on empathy would target men in the first group, but would probably have little effect on the men in the other group(s). These lines of argument make it clear that the question of the presence or absence of an interaction effect is not only of theoretical interest but also of practical importance and, therefore, needs more attention in future studies.

### Limitations and Future Directions

Despite the significant contributions of this study, several limitations should be acknowledged. Firstly, the cross-sectional design precludes causal inferences. Longitudinal studies are needed to establish the temporal relationships between empathy, dominance/nurturance, hostile masculinity, impersonal sexuality, and sexual aggression. Ideally, the operationalization of hostile masculinity should represent all components of this heterogeneous construct and not only hostility toward women as in the present study.

Secondly, the sample covered a broad age range but was restricted to heterosexual men. It would be beneficial to ascertain whether a differential pattern of results is evident when comparing younger and older men or more diverse populations, including individuals from different cultural backgrounds and those with varying sexual orientations (e.g., Waldis et al., 2023). Furthermore, the sample size was relatively small. However, a robust statistical model was used to account for the complexity of the data. Additionally, while the use of latent regression models addressed some issues related to measurement error, further research is needed to refine these models and validate the constructs involved.

In conclusion, this study advances our understanding of the confluence model of sexual aggression by underscoring the critical role of empathy, whereas dominance/nurturance shows a negligible effect on sexual aggression. Hostile masculinity and impersonal sexuality remain key risk factors and empathy emerges as a potent protective factor that can mitigate the risk of sexual aggression. Although we could not confirm a moderating role for empathy on the effect of hostile masculinity and impersonal sexuality on sexual aggression, findings underscore the importance of incorporating empathy-enhancing components in prevention and intervention programs and highlight the need for continued research to explore the complex interplay of factors contributing to sexual aggression.

## Supplementary Information

Below is the link to the electronic supplementary material.

Supplementary file1 (DOCX 317 KB)

## Footnotes

1 The smallest possible value is zero, and only integers can be observed.

2 Interpretation note for latent variable negative binomial model: The parameter estimates from the latent variable negative binomial regression model indicate the influence of latent variables (constructed from multiple observed items) on the log count of the outcome variable. For example, the coefficient for the latent variable “Hostile masculinity” (0.604) indicates that for each unit increase in the latent construct, the log count of the dependent variable increases by 0.604. When exponentiated, exp(0.604) = 1.83, which corresponds to an 83% increase in the expected count of the outcome for each unit increase in the latent variable, holding other variables constant.

3 Following an anonymous reviewer’s advice, we recomputed Model 1 twice considering only the dominance or the nurturance scale, respectively, but not their difference. Both models led to a similar result as the one reported for their (latent) difference. However, when including only a latent dominance variable, the effect of the three-way interaction with hostile masculinity and impersonal sexuality on sexual aggression yielded statistical significance (for more details, see Supplementary Material). As this was a post hoc test and our expectations and hypotheses were focused mainly on the overlap nurturance and empathy, we refrain from a more detailed interpretation of this effect before a successful replication in a future study.

4 We would like to thank one of the three reviewers for this advice.
