# Supplementary material for: The Critical Role of Empathy Over Dominance/Nurturance in Predicting Sexual Aggression in the Confluence Model
Source: Arch Sex Behav. 2026 Sep 4;55(6):2627–41. doi: 10.1007/s10508-026-03510-5 (PMC13597548; doi:10.1007/s10508-026-03510-5)
Supplement: Supplementary file 1 [file 10508_2026_3510_MOESM1_ESM.docx]

Figure 1. Q-Q Plot of Residuals

Figure 2. Effect plots for the predictors in the negative binomial regression model 2 (final model)

Figure 3. Plot of residuals vs. predicted values
